# Surface-Enhanced Raman Scattering in Molecular Junctions

**DOI:** 10.3390/s17081901

**Published:** 2017-08-18

**Authors:** Madoka Iwane, Shintaro Fujii, Manabu Kiguchi

**Affiliations:** Department of Chemistry, Tokyo Institute of Technology, 2-12-1 Ookayama, Meguro-ku, Tokyo 152-8551, Japan; maasan017@gmail.com

**Keywords:** surface-enhanced Raman scattering, molecular electronics, single-molecular junction, electron transport

## Abstract

Surface-enhanced Raman scattering (SERS) is a surface-sensitive vibrational spectroscopy that allows Raman spectroscopy on a single molecular scale. Here, we present a review of SERS from molecular junctions, in which a single molecule or molecules are made to have contact from the top to the bottom of metal surfaces. The molecular junctions are nice platforms for SERS as well as transport measurement. Electronic characterization based on the transport measurements of molecular junctions has been extensively studied for the development of miniaturized electronic devices. Simultaneous SERS and transport measurement of the molecular junctions allow both structural (geometrical) and electronic information on the single molecule scale. The improvement of SERS measurement on molecular junctions open the door toward new nanoscience and nanotechnology in molecular electronics.

## 1. Introduction

Molecular junctions, where a small number of molecules bridge metal electrodes, have been envisioned as components for miniaturized electronic circuits since the 1970s. Aviram et al. first proposed theoretically that electrical rectification was possible with a molecular junction where a donor π system is bound to an acceptor π system via a σ bonded tunneling bridge [1]. In 1997, Metzger et al. experimentally showed the electrical rectification in the hexadecylquinolinium tricyanoquinodimethanide (C_16_H_33_Q-3CNQ) molecular junction [2]. At present, various functionalities including diode, transistor, and switch have been reported for single-molecular junctions [3,4,5,6,7,8,9,10]. Song et al. reported the transistor characteristics of alkanedithiol and benzene dithiol (BDT) single-molecule junctions [3]. The molecular currents were modulated by more than two orders of magnitude along with controlling the gate voltage between ±3 V. A single molecular diode was reported by Díez-Pérez et al. in 2009 [6]. They prepared a single molecular diode with the diblock dipyrimidinyldiphenyl molecule using the scanning tunneling microscope-break junction (STM-BJ) technique. The orientation of the asymmetric molecule was controlled through a selective deprotection strategy. The average rectification ratio at a 1.5 V bias was approximately five to one from positive to negative bias polarities. Kiguchi et al. reported single molecular resistive switch with the covered oligothiophene (QT) molecules [4]. The QT molecule has two same anchors (i.e., thiophene rings) at both the termini. The QT single-molecule junction showed three distinct conductance states depending on the gap size. The conductance was tuned by controlling the anchoring positions in the same molecule with the gap distance. 

Although various functionalities have been reported for molecular junctions, molecular devices are still far away from practical application, due to large variability of the device performance. For example, several different values (0.1 *G*_0_ (*G*_0_ = 2 *e*^2^/*h*), 0.01 *G*_0_, 4 × 10^−4^ *G*_0_) were reported as conductance of a benzenedithiol (BDT) single-molecular junction [3,11,12,13,14]. One of the origins of the variability of the charge transport property is due to the lack of the direct structural information of the single-molecular junction. Theoretical study showed that the conductance of the BDT single-molecular junction depended on the adsorption geometries on Au electrodes. The calculated conductance was 0.078 *G*_0_, 0.040 *G*_0_, and 0.004 *G*_0_ for bridge, hollow, atop site, respectively [13]. Generally, only electrical conductivity of the single-molecular junctions has been discussed without structural and electronical characterization of the molecular junctions. 

Vibrational spectroscopy is the most straightforward method to determine the atomic and electronic structure of the molecular junction because it provides a molecular fingerprint that can be used to identify the bridging molecule and molecular adsorption site. In 2002, point-contact spectroscopy (PCS) was first exercised to the hydrogen single-molecular junction [15]. PCS observed a small conductance change caused by the electron—phonon interaction of the single-molecular junction. Peaks in *d*^2^*I/dV*^2^-*V* curves provide the vibrational energy of the single-molecular junction, which unambiguously clarifies the existence of the molecule between metal electrodes. The PCS and inelastic electron tunneling spectroscopy (IETS) have been employed for numerous molecular junctions and were established as standard methods to identify the molecular junctions [16,17,18]. However, there is a drawback in these spectroscopies. The peak width in the spectrum increases with the temperature, and we can measure PCS and IETS only at low temperature (~4 K). For practical application, it is essential to measure the vibrational spectroscopy at room temperature.

Optical spectroscopic techniques, such as IR and Raman spectroscopy, are promising for vibration spectroscopy of the molecular junctions at room temperature. However, it seems difficult to use optical spectroscopes to observe the molecular junction. First, it is not easy to focus light to single-molecule size; second, the signal from a single or few molecules is too weak to detect. Fortunately, surface-enhanced Raman scattering (SERS) can overcome these difficulties thanks to the enhanced field formed between metal electrodes [19,20,21,22,23,24,25]. When light is irradiated onto metal nano electrodes whose size is smaller than the light wavelength, localized surface plasmon is excited in the metal nano electrodes. As the metal nano electrodes approach each other, the localized plasmon modes hybridize, resulting in a strong electric field between metal nano electrodes. This enhanced field increases the Raman signal. Since Raman scattering is a second-order optical process, the metallic nanostructure acts as an amplifier for both the incoming and scattered wave fields. The SERS enhancement factor can be as large as 10^15^ in the metal nanogap, which is sufficient to study Raman scattering of a single molecule [26,27,28,29]. SERS measurement of a single molecule was first demonstrated using random aggregates of colloidal Ag nanoparticles with crystal violet and rhodamine 6 G molecules. In the nano particle system, the molecules bridge metal nano structures, so this system can regarded as molecular junctions. The SERS of the molecule in the molecular junction could be detected thanks to the similar enhancement mechanism. In SERS measurement of molecular junctions, simultaneous SERS and conductance measurement are crucial. The conductance of the junction provides the information about the molecular junction; gap size, the number of the bridging molecules in the junction, and so on. The complementary information is obtained by SERS and conductance measurements. In this review article, we discuss the SERS studies on the molecular junctions.

## 2. SERS Measurement on the Molecular Junction 

**Figure 1:**
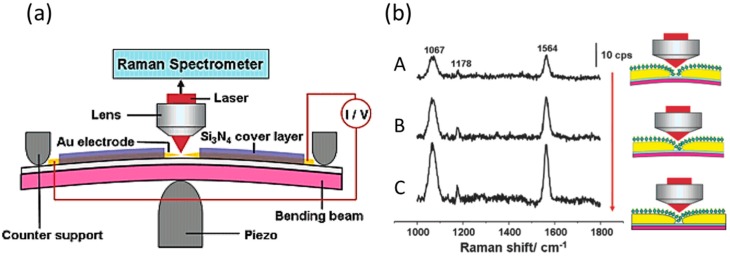
(**a**) The experimental setup for surface-enhanced Raman scattering (SERS) of molecular junctions fabricated with a mechanically controllable break junction (MCBJ); (**b**) SERS of 1,4-benzenedithiol (BDT) molecular junctions with different gap widths. The gap width was (A) 0.8 nm, (B) 0.6 nm, and (C) 0.4 nm. (*λ*_ex_: 632.8 nm) [30].

In 2006, the first SERS study of the molecular junction was reported by Tian et al. [30]. The BDT molecular junction was prepared by mechanically controllable break junction (MCBJ). In the MCBJ technique, a metal wire is attached on a flexible substrate. Target molecules are adsorbed on the surface of metal by immersing the wire in the solution containing the molecules. The sample is mounted in a three-point bending configuration. By bending the substrate, the metal wire is mechanically broken, leaving two atomically sharp electrodes separated by a nanogap. Molecules diffuse on the metal wire, and finally molecules are trapped in the nanoscale gap, forming molecular junctions [31,32]. Using the experimental setup shown in Figure 1a, the authors observed SERS enhancement only at the gap. The SERS signals from the smooth surface and edge of electrodes were weak. The polarization dependence of incident light on SERS supported the molecular junction and gave significant SERS signals. Strong SERS signals were detected when the polarization of the incident laser was parallel to the junction axis. When the polarization of the incident laser was perpendicular to the junction axis, the SERS intensity decreased greatly. In the SERS of BDT molecular junction, strong peaks were observed at 1068 cm^−1^ and 1568 cm^−1^, which were assigned to a ring breathing mode (ν_1_) and C=C stretching mode (ν_8a_) of the BDT molecule, respectively. Other peaks were also assigned to the vibrational modes of the BDT molecule, which clearly showed the existence of the BDT molecules in the gap between the metal electrodes. The authors studied the dependence of SERS intensity as a function of the gap width. The gap width was evaluated by measuring the tunneling current across the gap. Figure 1b shows three SERS spectra of BDT molecules with different gap widths. The SERS intensity increased considerably when the gap width was decreased from 0.8 to 0.4 nm. This enhancement in SERS intensity was explained by the increase in electromagnetic field as one reduced the gap.

**Figure 2:**
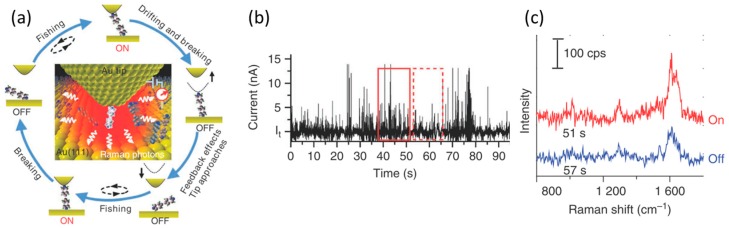
(**a**) Schematic view of the fishing-mode tip-enhanced Raman scattering (FM-TERS) for simultaneous measurement of conductance and TERS for single-molecular junctions; (**b**) Time course of conductance for the Au scanning tunneling microscopy tip/4,4′-bipyridine/Au (111) system; (**c**) TERS spectra corresponding to the ON and OFF states. (*λ*_ex_: 632.8 nm, laser intensity: 5 mW) [33].

The first SERS of the single-molecular junction was reported by Liu et al. in 2011 [33]. They measured SERS of the single-molecular junction with the fishing-mode tip-enhanced Raman scattering (TERS). The fishing-mode TERS (FM-TERS) is achieved by combining ‘fishing-mode’ STM (FM-STM) with TERS. In the FM-STM mode (Figure 2a), the proportional gain and the integral gain are decreased in order to decrease the response of the STM feedback. The current (conductance) though the STM junction is continuously monitored. When a molecular junction is formed, the electric current increases. The STM tip is retracted to decrease the current under the low-feedback condition. As the STM tip is moved away from the surface, the molecular junction breaks and the electric current decreases. The low-feedback system forces the tip to approach to the surface to regain the tunneling current. Figure 2b shows the time sequence conductance curve for 4,4′-bipyridine (BPY) in the junction between a Au STM tip and a Au substrate. Current jumps were observed and the time scale of each jump was a fraction of a millisecond. These current jumps were attributed to the formation (ON) and breaking (OFF) of the BPY single-molecular junction. The TERS signal fluctuated when the electric conductance switched between the single-molecular junction (ON) state and the breaking (OFF) state, indicating that the change in the TERS signal originated from the single molecular events. The TERS signal from the ON state showed the increase in intensities and peak widths, and shifting of peak positions (Figure 2c). Clear C—C stretching mode was observed around 1620 cm^−1^ (C—N/C—C dephasing stretching mode; *ν*_8a_) in SERS, which confirmed that the BPY molecule bridged Au electrodes. 

**Figure 3:**
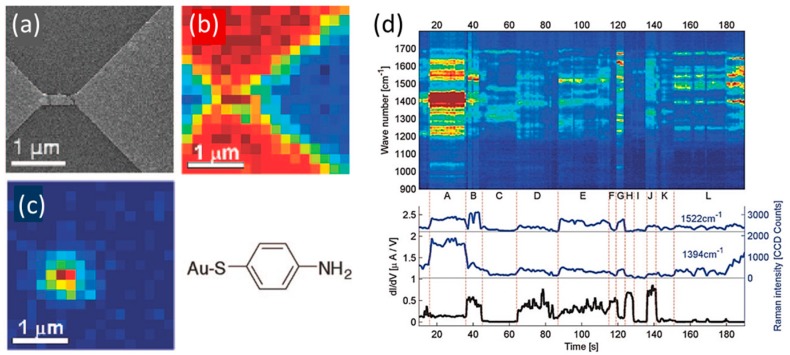
(**a**) Scanning electron microscope (SEM) image of the sample; (**b**) Map of the substrate Si 520 cm^−1^ peak; (**c**) Map of the p-mercaptoaniline (pMA) SERS signal of 1590 cm^−1^ (a_1_ symmetry mode); (**d**) Waterfall plot of SERS and conductance for a pMA molecular junction (λ_ex_: 785 nm, laser intensity: 0.5 mW) [34].

Stable molecular junction could be fabricated with the fixed electrode on the flat substrate. The fixed electrodes could be prepared using electromigration (EM) technique [34]. The EM process originates from the momentum transfer from electrons to metal atoms, so called electron wind force, and/or Joule heating. When a metal wire is electrically heated, the metal atoms become mobile. Because the momentum transfers from electrons to metal atoms, the atoms move in the opposite direction of current flow. The EM process is accomplished by ramping a voltage across the metal wire while monitoring the current. As the current increases, the conductance starts to change. Upon further increase of the current, the conductance drops suddenly to almost zero, and the metal wire breaks, forming the nanoscale gap. Although the number of the bridging molecules between the fixed electrodes cannot be controlled, mechanical stability of the electrodes is much higher than those fabricated by MCBJ, FM-STM, and other break junction techniques. Figure 3a is the scanning electron (SEM) image of the fixed electrodes fabricated on a Si substrate, and on which p-mercaptoaniline (pMA) was deposited from solutions [34]. Figure 3b is the spatial map of the Si 520 cm^−1^ peak in the same region shown in Figure 3a. The Si peak was not observed on the Au electrodes. Figure 3c shows the distribution of the pMA-SERS signal of 1590 cm^−1^ (a_1_ symmetry mode). The SERS signal was only observed at the nanogap. Figure 3d shows the example of the simultaneous conductance and the SERS measurements of a pMA molecular junction (a pMA molecule trapped in the Au electrodes). The rapid changes in the SERS signals correlated with conductance changes but the relationship was complicated. In some periods, the increases in SERS intensity correlated with increase in conductance, while increases in SERS intensity correlated with decrease in conductance in other period. The observed changes in conductance and SERS of molecular junction can be explained by the change in the atomic configuration of the molecular junction.

**Figure 4:**
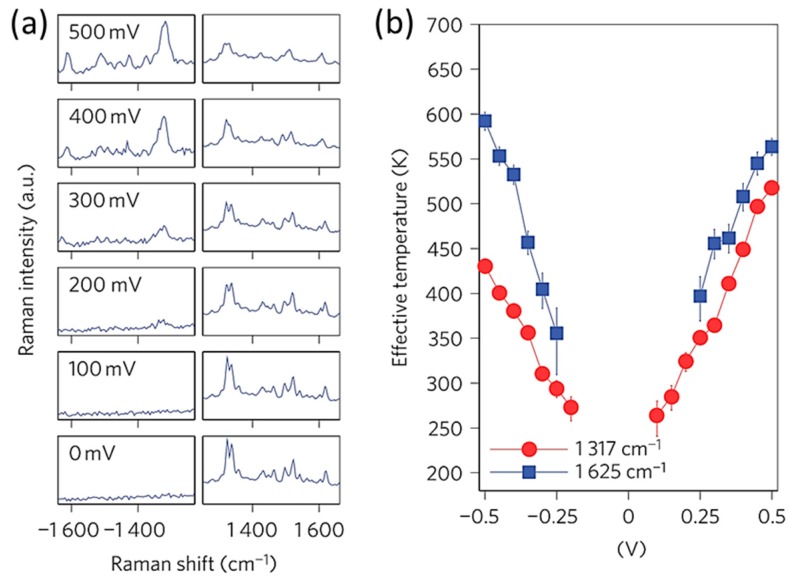
(**a**) Sample Raman spectra of the three-ring oligophenylene vinylene (OPV3) molecular junction. The full scale of the anti-Stokes and Stokes signal are 235 counts and 10,000 counts, respectively; (**b**) Effective temperature of the molecular junction as a function of bias voltage: 1317 cm^−1^ (red) and 1625 cm^−1^ (blue) [35].

Using a highly stable molecular junction with fixed electrodes, the temperatures of the charge-transporting molecular junctions were investigated by Ward et al. and Ioffe et al. [35,36]. They evaluated the effective temperature ($T_{\nu }^{eff}$) of the molecular junction for each vibrational mode by the Stokes (S) and the anti-Stokes (AS) ratio [35,36]. Here, $T_{\nu }^{eff}$ is represented by

(1) $$\frac{I_{\nu }^{AS}}{I_{\nu }^{S}}=A_{\nu }\frac{{\left(\varpi_{L}+\varpi_{\nu }\right)}^{4}}{{\left(\varpi_{L}-\varpi_{\nu }\right)}^{4}}\exp\left(-\hbar \varpi_{\nu }/k_{B}T_{\nu }^{eff}\right)$$

where $I_{\nu }^{S}$ and $I_{\nu }^{AS}$ are the intensity of the Stokes and anti-Stokes Raman mode, $\varpi_{L}$ and $\varpi_{\nu }$ are the frequency of the incident laser, wave number of the vibrational mode, and $A_{\nu }$ is a correction factor, respectively [35]. Figure 4a shows the bias voltage dependence of SERS for the three-ring oligophenylene vinylene (OPV3) molecular junction. The intensity of the anti-Stokes SERS increased with the voltage. Figure 4b shows the $T_{\nu }^{eff}$ for different modes as a function of bias voltage. The $T_{\nu }^{eff}$ linearly increased with the bias voltage. Although the effective temperature varied with the vibrational modes, generally speaking the effective temperature can increase by several hundred by application of the bias voltages with a few hundred millivolts.

## 3. Correlation between SERS and Atomic Structure of the Molecular Junction

**Figure 5:**
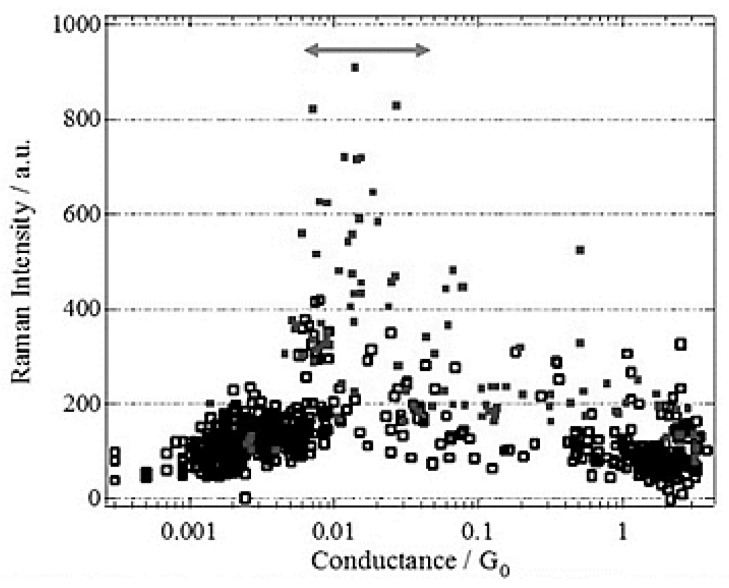
SERS intensity as a function of the conductance of the BPY molecular junction. The arrow indicates the conductance regime of BPY single-molecular junction [37]. The hollow and solid squares correspond to the molecular junctions showing the totally symmetric *a* mode and the non-totally symmetric *b* mode in SERS, respectively.

Raman shift provides us the information about the atomic structure of the molecular junction. Konishi et al. observed the structural change of the BPY single-molecular junction as a change in the SERS spectra [37]. First, they confirmed that the SERS signal originated from the single-molecular junction by the correlation between SERS intensity and electric conductance. Figure 5 shows the SERS intensity as a function of conductance of the BPY molecular junctions fabricated with nano MCBJ electrodes. A significant increase in SERS intensity was observed around 0.01 *G*_0_. Previous research revealed that the conductance of the BPY single-molecular junction was 10^−2^ *G*_0_ [38], indicating the single molecular origin of the SERS spectrum.

**Figure 6:**
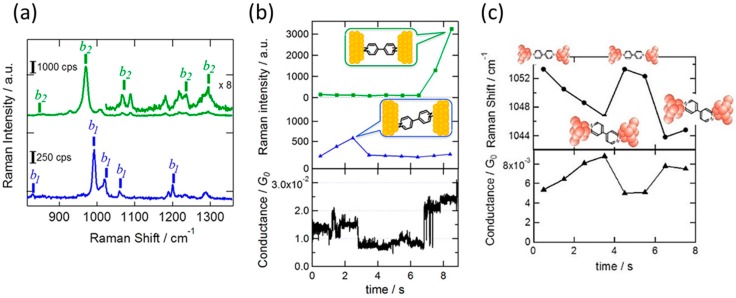
(**a**) Two types of SERS spectra of the BPY single-molecular junctions (λ_ex_: 785 nm, laser intensity: 0.5 mW); (**b**) Time evolution of the Raman intensity of the *b*_1_ mode and *b*_2_ mode, and the conductance of the BPY single-molecular junction; (**c**) Time course of the Raman shift of the ring breathing mode around 1050 cm^−1^ and the conductance of the BPY single-molecular junction [37].

Typically, two types of SERS spectra were observed for the BPY single-molecular junction (Figure 6a). The bands at 840, 998, 1026 and 1205 cm^−1^ (bottom of Figure 6a) were assigned to the non-totally symmetric *b*_1_ mode. The band at 975 cm^−1^ (top of Figure 6a) was assigned to the non-totally symmetric *b*_2_ modes. The theoretical calculation showed that the b_2_ mode appeared when the BPY molecule vertically bridged the gap, and the *b*_1_ mode appeared when the molecule became tilted. The conformational change of the single-molecular junction was observed as a change in SERS spectra. Figure 6b depicts time course of the conductance and the intensity of *b*_1_ and *b*_2_ mode in the SERS spectra of the BPY single-molecular junction. The *b*_1_ mode was detected until 3 s, and the conductance was higher than 0.01 *G*_0_. At 4 s, the *b*_1_ mode disappeared together with the rapid decrease in conductance below 0.01 *G*_0_. At 7 s, the *b*_2_ mode appeared and conductance recovered to the initial value. The observed SERS spectra indicated that the BPY molecule initially bridged the metal electrodes with its molecular long axis inclining to the junction axis, after which the molecular junction broke (3 s), and finally the molecular bridges the electrodes with its molecular long axis parallel to the junction axis.

As well as the dramatic change in the atomic configuration of the junction, a slight molecular orientation change was detected as a change in the Raman shift and conductance of the BPY single-molecular junction. Figure 6c shows the time course of electric conductance and the wavenumber of the ring breathing mode around 1050 cm^−1^ in the single-molecular junction regime. The wavenumber of the ring breathing-mode increased (decreased) when the conductance decreased (increased). The observed anticorrelation between wavenumber and electric conductance could be explained based on the metal—molecule interaction. When a molecule adsorbs on the metal surface, the highest occupied molecular orbital (HOMO) of BPY hybridizes with the metal unoccupied states, and the lowest unoccupied molecular orbital (LUMO) hybridizes with the metal occupied states. These two interactions lead to the strong molecule—metal bond. Through these interactions, electrons are removed from the HOMO (bonding) and are injected into the LUMO (anti-bonding). Both electron transfers weakens the bonds of molecule itself. The degree of decrease in the molecular bond strength, corresponding to the wavenumber, depends on the strength of the interaction between metal and molecule. An increase in the metal–molecule interaction, thus, leads to a decrease in the energy of the wavenumber of the molecule. Meanwhile, the electric conductance of the single-molecular junction is described as:

(2) $$G=\frac{2e^{2}}{h}\frac{4\Gamma^{2}}{\Delta^{2}+4\Gamma^{2}},$$

in the single-level tunneling model. Here *Γ* and *Δ* are the electric coupling between the metal and molecular orbitals and the energy difference between the metal and molecular orbitals, respectively. The electric conductance increases with the electric coupling, that is, the metal—molecule interaction. The increase in the metal—molecule interaction, thus, causes the increase in the electric conductance of the single-molecular junction, and decrease in the wavenumber, as experimentally observed in Figure 6c. The fluctuation of the wavenumber synchronized with the change in electric conductance directly showed the dynamic motion of the single-molecular junction.

**Figure 7:**
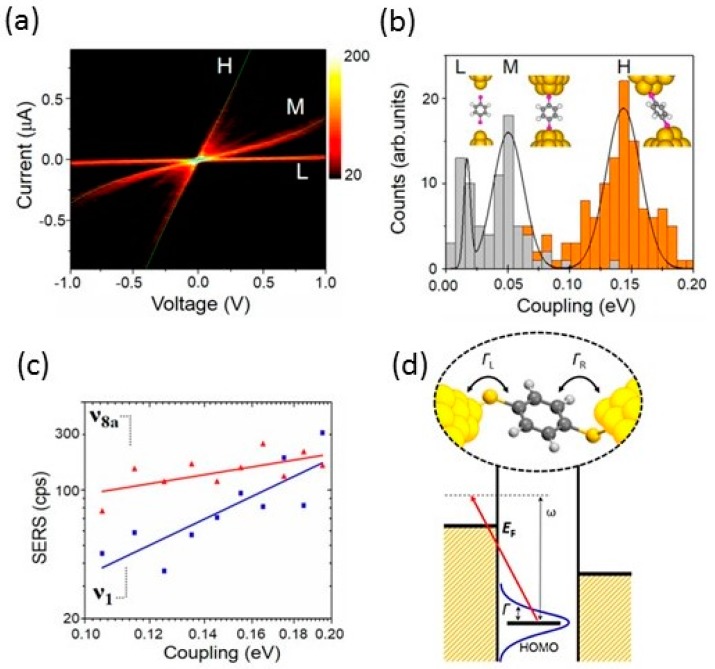
(**a**) Bidimensional *I*—*V* histogram summarizing the individual *I*—*V* response of single-molecule BDT junctions; (**b**) Statistical distribution of *Γ*. Orange counts correspond to ν_8a_-active samples; (**c**) Correlation between the average intensity of the SERS signal (*ν*_1_ and *ν*_8_ modes) as a function of *Γ* on a log—log plot; (**d**) Photo-induced charge transfer transition from HOMO to metal unoccupied state. The discrete molecular level is broadened by *Γ* [42].

In the above studies, the atomic structure of the single-molecular junction is argued by considering the electric conductance and vibrational energy in SERS. The current—voltage (*I*—*V*) response provides much more information than conductance (e.g., electronic structure, metal—molecule interface structure of the molecular junction). Assuming that electron transport through a single channel, *I*—*V* response is represented as

(3) $$I(V)=\frac{2e}{h}\Gamma \left\{\tan^{-1}(\frac{\frac{1}{2}eV-\varepsilon_{0}}{\Gamma })+\tan^{-1}(\frac{\frac{1}{2}eV+\varepsilon_{0}}{\Gamma })\right\}$$

where *ε*_0_ is the energy of the conduction orbital [39,40,41]. The parameters, *ε*_0_ and *Γ*, are sensitive to the metal—molecule interface structure of the single-molecular junction. By fitting the *I*–*V* response to Equation (3), *ε*_0_ and *Γ* can be obtained. Figure 7a shows the distribution of *I*–*V* responses for BDT single-molecular junctions. Three statistically high-probable nonlinear curves were clearly observed (H, M and L). The distribution of strength *Γ* also indicates probable three *Γ*’s in the *Γ* histogram (Figure 7b) [42]. By comparing the experimentally obtained *ε*_0_, *Γ*, and conductance with the calculated ones, the experimentally observed H, M and L states were assigned to the bridge, hollow, and top adsorption site geometry, respectively.

Then, the SERS of the BDT single-molecular junction is discussed based on the results obtained by *I*—*V* responses. Orange counts in the *Γ* histogram (Figure 7b) corresponds to SERS active samples. The counts were found to concentrate in the H state (bridge). In other words, we can conclude that the BDT molecule occupies the bridge site when its SERS signal is detected. Moreover, the SERS intensity was found to increase with *Γ*. Figure 7c shows the relationship between the intensity of the SERS signal (*I*_s_) and *Γ* on a log—log plot. The observed distribution clearly corresponded to a power law relationship, *I*_s_ ∝ *Γ^α^*. This is the first in-situ study of the correlation between the optical and electronic properties in single-molecular junctions [42].

The site selectivity and power law relationship were explained by the SERS enhancement mechanism (Figure 7d). The SERS signal gains intensity from two contributions: electromagnetic (EMM) and chemical (CM) effects [21,22,43,44]. The EMM effect originates from local field enhancement. Although the EMM effect was the major contributing factor in SERS of the BDT single-molecular junction, the site selectively and power law relationship could be explained by the CM effect. One of the main sources for the CM effect is charge transfer resonance taking place between metal states near the Fermi level and molecular electronic stats (from HOMO to metal unoccupied state or metal occupied state to LUMO) [45]. The charge transfer between the metal and the molecule easily occurs when the metal—molecule interaction is strong. Therefore, the SERS intensity increases with *Γ*. The theoretical calculation based on a single-level Anderson model reproduced the power law relationship. As for the site selectivity, electronic coupling was small for the hollow and atop, and thus, the SERS intensity corresponding to these sites were under the detection limit (SERS inactive). Only high coupling state (H) is visible, which causes site selectivity.

## 4. Effect of Light Irradiation and Bias Voltage Application on Molecular Junction

**Figure 8:**
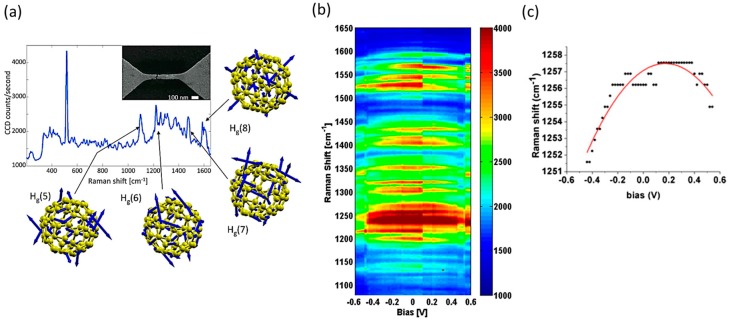
(**a**) Example of SERS of the C_60_ molecular junction fabricated with the electron migration technique. Inset: SEM image of the electrode. Surrounding figures illustrate vibrational modes; (**b**) SERS of the C_60_ molecular junction as a function of the bias voltage; (**c**) Raman shift as a function of the bias voltage for a particular mode: 1258 cm^−1^ [46].

In the above discussion, we assume that the effect of laser irradiation and bias voltage application on the molecular junction is negligibly small. Although the bias voltage-induced heating was observed for OPV3 molecular junction [35], it is still assumed that the atomic structure of the molecular junction was not changed by the application of bias voltage. However, the light irradiation and application of bias voltage can affect the molecular junction. Li et al. reported the bias-driven Raman shift for the C_60_ molecular junctions using the fixed electrode, which were fabricated using EM technique [46]. Figure 8a shows the SERS of the C_60_ molecular junction. The sharp peak at 520 cm^−1^ is from the underlying Si substrate, and the peaks between 1000 cm^−1^ and 1600 cm^−1^ are from vibrational modes of C_60_ in the junction. Figure 8b shows the SERS of C_60_ molecular junction as a function of the bias voltages in the bias regime from –0.6 to 0.6 V. Many of the vibrational modes shifted toward low energies when the bias voltage increased. This bias-driven shift is apparent as a curvature of the spectral features (Figure 8b). The bias driven shifts can be represented by δω~*V*^2^. This quadratic dependence on the bias voltage is clearly seen in Figure 8c, which shows the Raman shift as a function of the bias voltage for a particular mode of 1258 cm^−1^. They discussed that the C_60_ charge state was changed by the application of the bias voltage, and the change in the charge state caused the vibrational energy shifts of the C_60_ molecular junction. By applying the bias voltage across the junction, electron(s) can be injected to the closer lying LUMO and the addition of an electron to the antibonding LUMO softens intramolecular bonds. 

**Figure 9:**
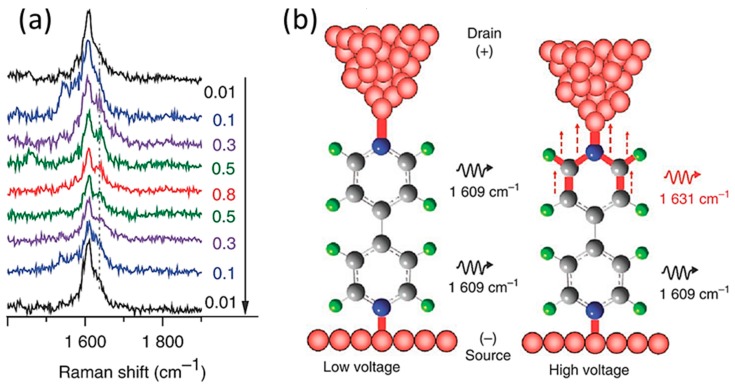
(**a**) The bias voltage dependence on TERS of BPY molecular junction featuring the *ν*_8a_ band (1610 cm^−1^); (**b**) The schematic of the BPY single-molecular junction at a low and high bias-voltage (*λ*_ex_: 632.8 nm, laser intensity: 5 mW) [33].

The shift of the SERS peaks was observed for the BPY single-molecular junction fabricated with the FM-STM [33]. Figure 9 shows the bias voltage dependence of SERS of the BPY single-molecular junction. When the bias voltage was increased from 10 to 800 mV, one peak around 1610 cm^−1^ (*ν*_8a_) changed to double peaks above 100 mV. When the bias voltage was reversed, the peak splitting disappeared, indicating that the peak splitting relates with the bias voltage. The BPY molecule consists of two pyridine rings. The theoretical calculation revealed that the increase in the bias voltage lowers the Fermi level and increases the density of electric charge on the Au tip. This led to an increase in the strength of the chemical bond between the Au tip and the pyridine ring in contact with it. The peak split could be understood in terms of the different bonding interactions between the Au tip and BPY and between BPY and the Au.

**Figure 10:**
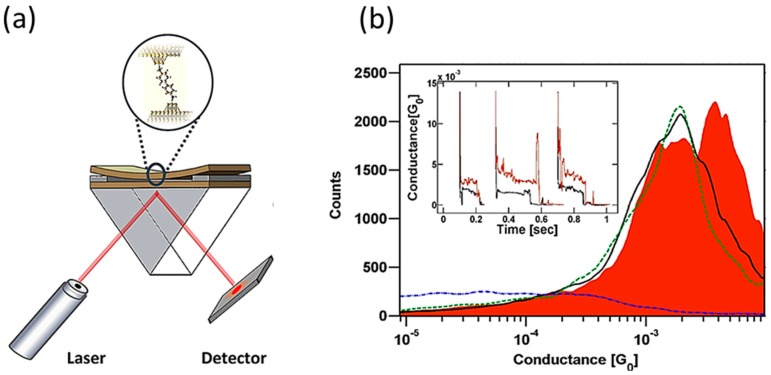
(**a**) Schematic view of squeezable break junction setup for conductance measurement of the molecular junction; (**b**) Conductance histograms of the 2,7-diaminofluorene molecular junction measured without (black curve) and with (red colored area) continuous wave illumination (wavelength: 781 nm, power: 10 mW). Inset: Examples of the conductance traces of the 2,7-diaminofluorene molecular junctions without (black) and with (red) laser illumination [47].

As well as the bias voltage, the light irradiation can affect the electric conductance through the single-molecular junction. Vadai et al. reported the plasmon-induced conductance enhancement in the single-molecular junction [47]. They fabricated single-molecular junctions with a squeezable break junction (SBJ) technique (Figure 10a). The SBJ consists of two Au electrodes evaporated on top of 1 mm thick glass slides. The gap between Au electrodes was mechanically controlled by applying a squeezing force against the top slide. The wavelength of the light was 781 nm and its power was 10 mW. Figure 10b shows the conductance histogram of 2,7-diaminofluorene (DAF) molecular junction measured without and with laser illumination. The conductance of the single DAF-molecular junction was 1.9 × 10^−3^ *G*_0_ without laser illumination. Under the irradiation of light, a new high conductance peak appeared in the conductance histogram at 3.7 × 10^−3^ *G*_0_ in addition to the characteristic low conductance peak at 1.9 × 10^−3^ *G*_0_. The low conductance peak (1.9 × 10^−3^ *G*_0_) did not entirely shift to a high conductance value (3.7 × 10^−3^ *G*_0_) and the both low and high conductance peaks were observed under the light illumination. The authors explained this observation in the following way. In each measurement of a conductance trace, local hot spots can be formed on the surface of Au electrodes where the localized surface plasmon is excited. The formation of local hot spot is sensitive to local roughness of the surface, and the propagation length (*L*_p_) of localized surface plasmon is about a few 10 nm. The localized plasmon affects the conductance of a single-molecular junction only if the distance between the molecule in the junction and hot spot is smaller than *L*_p_. Because this is not necessarily in the case of all conductance measurements, only a fraction of the measurements was affected by the localized surface plasmon. They explained the increase in conductance of the DAF single-molecular junction by taking the plasmon field as an oscillating potential *V*_ω_ at the plasmon frequency ω across the junction. The calculated transmission probability of the DAF single-molecular junction was dominated by HOMO (highest occupied molecular orbital) in the energy range of E_F_ − *ħ*ω < E < E_F_ + *ħ*ω. The magnitude of gate voltage (*V*_ω_) was evaluated to be 0.169 V.

## 5. Conclusions

In conclusion, we have reviewed the SERS studies of the molecular junction. The molecular junctions have been envisioned as components for miniaturized electronic circuits. The SERS is one of the promising tools to characterize the molecular devices during device operation at room temperature. In SERS measurement of the molecular junction, simultaneous SERS and electrical measurement are crucial. The complementary information is obtained by SERS and electrical measurements, which are essential to fully characterize the molecular junction. The simultaneous conductance and SERS measurement clarify the bridging of target molecule between metal electrodes, and effective temperature, dynamical motion of molecule in the gap, and so on. The simultaneous *I*—*V* response and SERS can clarify the atomic configuration at the molecule—metal interface. The correlated SERS measurements show selectivity towards one of the adsorption sites. Site-sensitivity represents a crucial step toward the reliable integration of millions of molecular components into a working device. The development of the new measurement technique open the door to new science. The further development of the SERS measurement technique should reveal interesting phenomena and useful insight in nanosciece and nanotechnology.
